# Age-Related Variation in Health Status after Age 60

**DOI:** 10.1371/journal.pone.0120077

**Published:** 2015-03-03

**Authors:** Giola Santoni, Sara Angleman, Anna-Karin Welmer, Francesca Mangialasche, Alessandra Marengoni, Laura Fratiglioni

**Affiliations:** 1 Aging Research Center, Department of Neurobiology, Care Sciences and Society, Karolinska Institutet and Stockholm University, Stockholm, Sweden; 2 Karolinska University Hospital, Stockholm, Sweden; 3 Section of Gerontology and Geriatrics, Department of Medicine, University of Perugia, Perugia, Italy; 4 Geriatric Unit, Department Clinical and Experimental Science, University of Brescia, Brescia, Italy; 5 Stockholm Gerontology Research Center, Department of Neurobiology, Care Sciences and Society, Karolinska Institutet, Stockholm, Sweden; University of Naples Federico II, ITALY

## Abstract

**Background:**

Disability, functionality, and morbidity are often used to describe the health of the elderly. Although particularly important when planning health and social services, knowledge about their distribution and aggregation at different ages is limited. We aim to characterize the variation of health status in a 60+ old population using five indicators of health separately and in combination.

**Methods:**

3080 adults 60+ living in Sweden between 2001 and 2004 and participating at the SNAC-K population-based cohort study. Health indicators: number of chronic diseases, gait speed, Mini Mental State Examination (MMSE), disability in instrumental-activities of daily living (I-ADL), and in personal-ADL (P-ADL).

**Results:**

Probability of multimorbidity and probability of slow gait speed were already above 60% and 20% among sexagenarians. Median MMSE and median I-ADL showed good performance range until age 84; median P-ADL was close to zero up to age 90. Thirty% of sexagenarians and 11% of septuagenarians had no morbidity and no impairment, 92% and 80% of them had no disability. Twenty-eight% of octogenarians had multimorbidity but only 27% had some I-ADL disability. Among nonagenarians, 13% had severe disability and impaired functioning while 12% had multimorbidity and slow gait speed.

**Conclusions:**

Age 80-85 is a transitional period when major health changes take place. Until age 80, most people do not have functional impairment or disability, despite the presence of chronic disorders. Disability becomes common only after age 90. This implies an increasing need of medical care after age 70, whereas social care, including institutionalization, becomes a necessity only in nonagenarians.

## Introduction

The older population is increasing worldwide,[1] a development that will challenge societies and their health care systems. The best way to face these challenges is to prolong the proportion of years of life lived in good health by identifying realistic preventive and care priorities.[2] An important step in this direction is to better understand the age-related changes in older adults and to detect the most prevalent patterns in the different phases of aging.

The older population consists of an extremely heterogeneous group of persons;[3] the older the age group, the greater the variation found in cognition, physical and sensory function, and social engagement, to mention just a few examples.[4] For that reasons, there is a large agreement among researchers and clinicians in using multiple health indicators to capture the complexity and variability of health status in older adults.[1] Most of the currently used indexes that objectively assess the general health status of older adults (e.g. comprehensive geriatric assessment and Multidimensional Prognostic Index[5] constructs to mention a few) include four dimensions:[6] morbidity, physical functioning, cognitive functioning, and disability (defined as dependence in Activities of Daily Living [ADL]). Although these indicators of poor health are correlated with each other and with survival,[7–10] knowledge about their distribution, aggregation in the general population, and occurrence at different ages is still very limited.

The aims of this study were to characterize the health status of 60+ old adults and to detect the age-related variability using 5objective health indicators. Specifically, we set out to explore the age-related differences between these indicators and to estimate the prevalence of the most frequent patterns of their aggregation.

## Methods

Data were gathered from the Swedish National study of Aging and Care in Kungsholmen (SNAC-K), a community-based longitudinal study of the general population in central Stockholm.[11] Participants were randomly selected from the population of adults aged 60+ living at home or in institutions in the Kungsholmen district of Stockholm between 2001 and 2004. To reduce attrition during follow-up, the sample was selected from 11 age cohorts: 60, 66, 72, 78, 81, 84, 87, 90, 93, 96, and 99+. The two youngest and the four oldest age groups were oversampled. Of the original 5111 people invited to participate, 521 were not eligible (200 dead, 262 without contact information: 59 deaf, moved away, or not Swdesh speaker). Among the remaining 4590, 1227 declined to participate, leaving a study population of 3363 (73% participation rate). In the present analyses, data were complete for 3080 participants.

Physicians made clinical diagnoses on the basis of the general health status of participants, laboratory tests, and hospital records. Diagnostic criteria were derived from the ICD10, except for dementia (DSM-IV), and diabetes. On the basis of a literature review and a previous report on multimorbidity,[12] a disease or a condition (i.e., the residual disability after an acute disease) was defined as chronic if it met one or more of the following criteria: was of prolonged duration; left residual disability; worsened quality of life; or required a long period of care, treatment, or rehabilitation. The number of chronic diseases (CD) occurring in the same person ranged from zero to ten.

Cognitive functioning was assessed with the Mini Mental State Examination (MMSE),[13] a measure of global cognitive decline that encompasses basic cognitive domains. MMSE score range was 0 to 30. Physical functioning was measured as gait speed. Participants were asked to walk 6m or, if the participant reported walking quite slowly, 2.4m. If the participant was unable to walk or attempted unsuccessfully to walk, a value of 0 was recorded. Gait speed range was 0–2m/sec. Disability was defined as the number of instrumental-ADL (I-ADL) and personal-ADL (P-ADL) the person was unable to perform independently. I-ADL measure the ability of the participant to live independently in the community. To avoid tasks that might be very gender specific, we included only 4 tasks in our analyses: grocery shopping, managing money, using the telephone, and using public transportation. We considered people who lived in an institution to be dependent in grocery shopping. P-ADL measure the ability to perform 5 basic self-care tasks: bathing, dressing, toileting, transferring, and eating.

### Ethics

SNAC-K received ethical permission for baseline and follow-ups from the Ethics Committee at Karolinska Institutet and the Regional Ethics Review Board in Stockholm (Dnrs: 01–114, 04–929/3, 2007/279–31). Written informed consent was obtained from all participants.

### Statistical analyses

In the statistical analyses, we accounted for the sampling design either by stratifying by or adjusting by age. In age-stratified analyses, 4 age groups were used: sexagenarian, septuagenarian, octogenarian, and nonagenarian (including centenarians). In age-adjusted analyses, age was modeled as a spline with 4 nodes.

Differences between participants and dropouts were analyzed with Fisher’s exact test. Risk ratios of non-participation were derived from generalized linear models (binomial distribution with log link) stratified by age and adjusted by sex and survival status since baseline (3 time intervals: alive after 6 years, deceased after 2 years, and deceased within 2 years).

To compute the association between age and each health indicator (number of CD, gait speed, MMSE score, I-ADL, and P-ADL) while adjusting by sex, we used logistic quantile regression[14]. For each outcome, we derived 10th percentile (p10), median, and 90th percentile (p90) curves to indicate the values below which 10%, 50%. and 90% of the population had better scores.

Logistic regression was used to derive the probability of poor health status across age, adjusted by sex. Several indicators of poor health status were considered: 1+ CD, gait speed<1.2 m/sec,[10] MMSE<27, MMSE<20, 1+ I-ADL disabilities, and 1+ P-ADL disabilities. For each indicator, we plotted the sex-adjusted probability curves as a function of age. Two cut-off points of MMSE were used to capture different levels of cognitive impairment.[15]

To explore the aggregation of different health indicators, we categorized each health measure into 2–3 groups: 3 groups for number of CD (0, 1, 2+), gait speed (<0.4, ≥0.4 and <1.2, and ≥1.2 m/sec);[10,16] and MMSE score (<20, 20–26, and >26); and 2 groups for both I-ADL and P-ADL (0, 1+). The cut-off of 1.2 for gait speed was the speed required for optimal community ambulation, and the cut-off of 0.4 was an indicator of severely impaired mobility.[10,16] Dementia was removed from the CD list because MMSE was present as a measure of cognitive status. Sixty-three different health combinations were present (“health states” in the manuscript). To estimate the prevalence of each health state, we ran a linear regression model adjusted by sex and stratified by age. We plotted the health states with prevalence >5%.

A sensitivity analysis of the effect of missing values was performed through imputations of ten new imputed datasets with multivariate imputation chained equation (MICE).[17]

Data analyzed with Stata/SE 13.0 (StataCorp LP., College Station, Texas, USA).

## Results

Of the 4590 participants alive and eligible at baseline, 1227 declined to participate. Participation rates were above 70% in all age groups and were similar among men and women (Table 1).

**Table 1:** Number and %s of participants and non-participants at the SNAC-K population study at baseline (years 2001 to 2004).

	Women		Men	
	Participants	Non-participants	Participants	Non-participants
Total, n (%)	2182 (73)	813 (27)	1181 (74)	414 (26)
Age group, n (%)				
Sexagenarians	735 (77)	223 (23)^a^	569 (76)	181 (24)
Septuagenarians	598 (72)	230 (28)	341 (73)	125 (27)
Octogenarians	448 (69)	204 (31)	186 (72)	73 (28)
Nonagenarians	401 (72)	156 (28)	85 (71)	35 (29)

^a^ p-value<0.01.

Analysis stratified by age and sex.

The proportion of people living in institution was significantly higher among participants (6%) than among non-participants (12 people, <1%) %), although it is possible the latter could be an underestimate, as there were some persons originally invited to participate who could not be contacted (N = 262). For the age cohorts 60 through 87, shorter time to death after the beginning of the study was associated with higher risk of non-participation (Table 2). This association was not present among the nonagenarians.

**Table 2:** Risk ratios (RR) and 95% confidence intervals (CIs) of being a non-participant by sex and vital status in one short and two longer time intervals.

	Sexagenarians (N = 1708)		Septuagenarians (N = 1294)		Octogenarians (N = 911)		Nonagenarians (N = 677)	
	N	RR (95% CI)	N	RR (95% CI)	N	RR (95% CI)	N	RR (95% CI)
Gender								
Men	750	ref. (1 00)	466	ref. (1.00)	259	ref. (1.00)	120	ref. (1.00)
Women	958	0.99 (0 84–1 17)	828	1.01 (0.84–1.20)	652	1.13 (0.90–1.41)	557	0.95 (0.70–1.29)
Survival status								
Alive^a^	1587	ref. (1 00)	1000	ref. (1.00)	499	ref. (1.00)	129	ref. (1.00)
Deceased after 2 years	91	1.13 (0 78–1 63)	211	1 26^b^ (1.01–1.58)	285	1.28^b^ (1.02–1.60)	299	1.25 (0.90–1.73)
Deceased within 2 years	30	2.86^c^ (2 14–3 82)	83	1 67^c^ (1.28–2.21)	127	1.73^c^ (1.34–2.22)	249	0.85 (0.59–1.24)

^a^ Within the first 6 years after the start of the study.

^b^ p-value < 0.05.

^c^ p-value < 0.001.

Analyses stratified by age. Data from the SNAC-K population study at baseline (years 2001 to 2004).

The complete dataset used in the analyses consisted of information about 3080 people (mean age 74 years; 64% women; 16% with <9 years of education). The 283 participants excluded because of missing data were significantly older (mean age 85), more likely to be women (75%), to have <9 years of education (30%), and to live in institution (23%) than the participants for whom data were complete.

**Fig 1:**
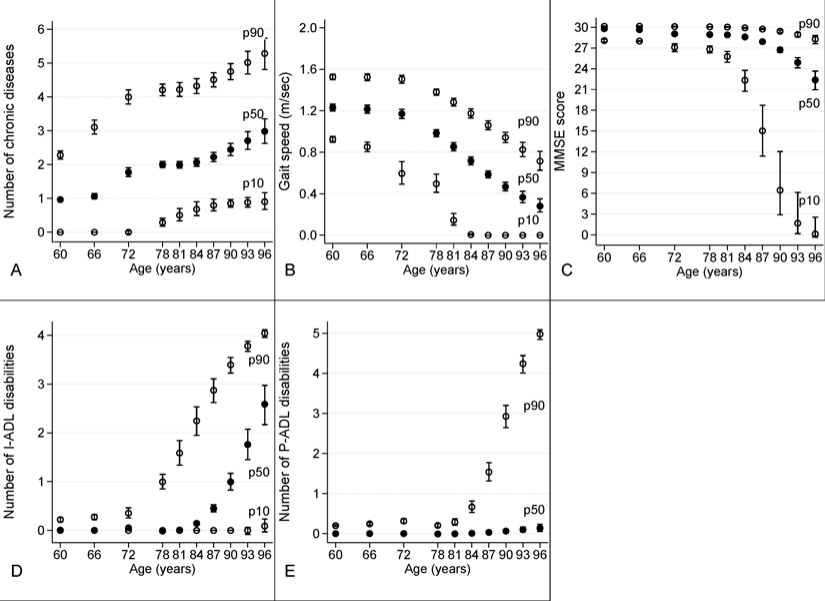
Distribution across age of A) number of chronic diseases; B) gait speed (m/sec); C) MMSE score; D) number of I-ADL disabilities; E) number of P-ADL disabilities. 10th percentile (p10, hollow circle), median (p50, full circle) and 90th percentile (p90, hollow circle) with relative 95% confidence intervals of the five health indicators adjusted by sex.

Fig. 1 shows p10, median, and p90 curves (95% CIs) as a function of age for number of CD, gait speed, MMSE score, number of I-ADL impairments, and number of P-ADL impairments. The three curves together give a graphical representation of the change of their distribution across age groups. Curves for MMSE, I-ADL, and P-ADL were all flat in the lower age range and worsened rapidly with increasing age. In contrast, both median and p90 of number of CD and of gait speed changed almost constantly with age. P90 of I-ADL started to increase in groups 72+, whereas median MMSE, I-ADL, and p90 of P-ADL started to change only in groups 81+. Additional analysis was performed by further adjusting for years of education as an indicator of socioeconomic status. The results were not different from the one presented in the paper (data not shown).

**Fig 2:**
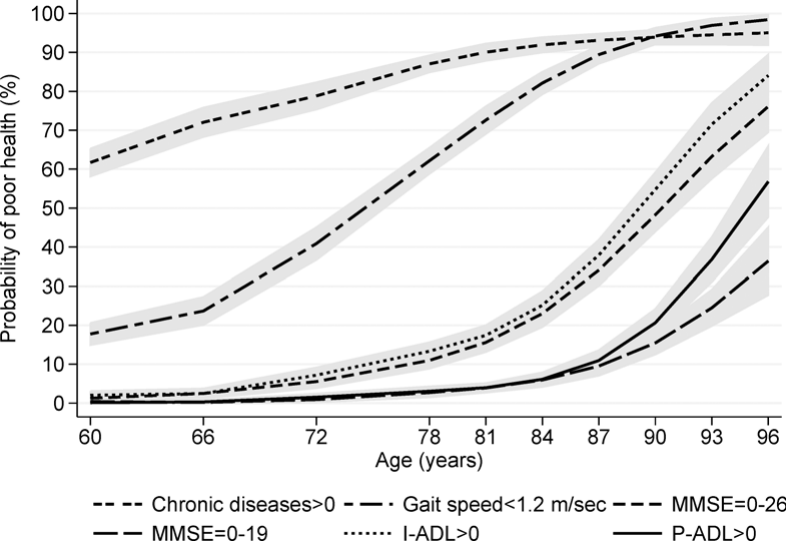
Sex-adjusted probability, per 100 persons, of poor health in one of the five health indicators as a function of age.

The analyses of the prevalence of impairment in each indicator reveled similarities in the age distribution of the indicators (Fig. 2). In particular, similar age-related changes were present in probability of I-ADL impairment and probability of any cognitive impairment (MMSE<27) and between probability of P-ADL impairment and probability of severe cognitive impairment (MMSE<20).

**Fig 3:**
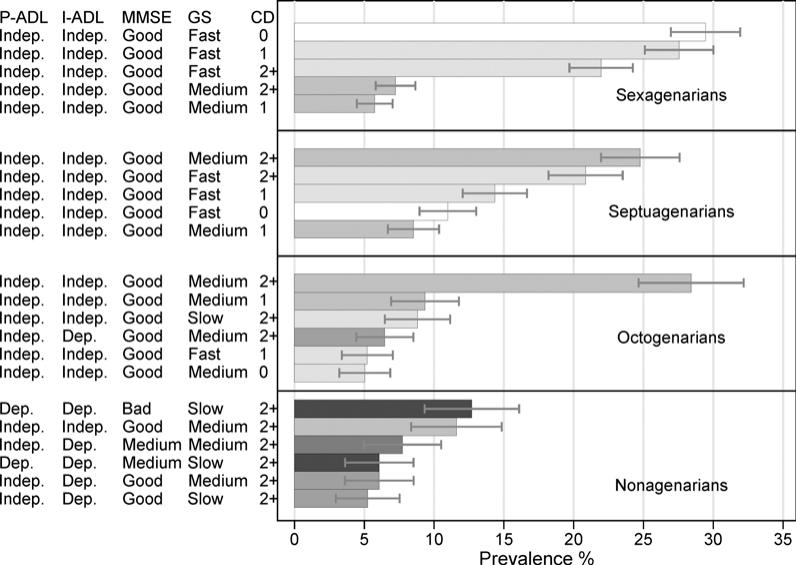
Sex-adjusted prevalence per 100 persons and 95% confidence intervals of health indicators and their aggregation by age. Only the most common (over 5%) indicators or their aggregations within each age group are reported. CD = number of chronic diseases. Gait speed (GS): slow = ≤0.4 m/sec; medium = 0.4–1.2 m/sec; fast: ≥ 1.2 m/sec. MMSE: good = ≥27; medium = 20–26; bad = <20.

Fig. 3 illustrates the prevalence of health states with figures over 5%, by age group. The best health state was characterized by people with no chronic diseases, gait speed equal or above 1.2 m/sec, MMSE score above 26, no I-ADL, and no P-ADL impairments. the prevalence of this state decreased with age, from 29% (95% CI: 26.97, 31.93) among the sexagenarians to 3% (95% CI: 1.48, 4.27) among the octogenarians. None of the nonagenarians belonged to this group. The most prevalent health states among people younger than age 80 were combinations of CD or of CD with mild impairment in gait speed. In this study population, the eighth decade of life was a transitional age, characterized by an increasing proportion of people with one or more I-ADL impairments. This increasing proportion pushed the percentage of people with a combination of at least one I-ADL disability and at least one another indicator of poor health beyond 5% of the total study population (6% with multimorbidity, slow gait speed, and 1+ I-ADL, 95% CI: 3.62, 8.53). Among nonagenarian, health status was characterized by a combination of multimorbidity, severe cognitive and physical impairment, and ADL disabilities. Furthermore, most of the common health states also included P-ADL disabilities. When health states with a prevalence of over 5% were summed together in each age group, they accounted for 92% of the sexagenarians and 80% of the septuagenarians. However, health states with a prevalence of over 5% accounted for only 63% of the octogenarians and 49% of the nonagenarians. Thus there was greater heterogeneity in the health states of the two oldest age groups.

Results of the analyses of the imputed data were similar to those of the analyses of the complete dataset; minor differences were present mostly among the oldest age groups.

## Discussion

In this large cohort, we were able to capture the complexity and heterogeneity of health status in 60+ old adults using five health indicators that can be easily implemented in clinical settings, including primary care. Until 80, most people do not have functional impairment or disability, despite the presence of morbidity or even multimorbidity. Disability is common only after age 90. The 80s are a transitional period when major health changes take place; often following the co-occurrence of more than one negative health event. These findings imply that at different ages different health indicators are better predictors of medical and social needs in older adults.

If we consider good health as the absence of chronic diseases, functional impairment, and disability, good health is still the most prevalent pattern among sexagenarians. However, even among octogenarians, the most prevalent health state is characterized by presence of chronic disorders with impairment only in gait speed. In other words, morbidity and multimorbidity start early in late adulthood, but functional dependence becomes common only for people older than age 90. Similarly, Jacobs et al.[18] showed that at age 70, health profiles were characterized by some multimorbidity with preserved cognitive and functional status that gradually deteriorated after 78. In the Newcastle 85+ study,[19] prevalence of disability was relatively low among 85-year-olds, whereas prevalence of 3+ diseases reached 90%.

Some health indicators shared similar age-related patterns. Similar tandem-slope patterns are present between any cognitive impairment and I-ADL disability and between severe cognitive impairment and P-ADL disability. Other studies have found a specific pattern of age-related increases in cognitive and physical decline that roughly parallel an increase in disability.[20,21] Our findings confirm that I-ADL disabilities are good indicators of initial cognitive impairment, and P-ADL disabilities are strongly related to dementia.[22] Among younger old people, gait speed seemed directly associated with morbidity, whereas older groups exhibited decreasing gait speed independently of chronic diseases. This finding is in line with reports in the literature,[8,23] showing the association between limitations in physical functioning and chronic diseases is less evident among the oldest old than among younger old adults. The negative relationship between age and gait speed might reflect the decrease in muscle mass that starts around age 50 and that can lead to sarcopenia.[24] In our population gait speed started to decline even before the presence of any disability confirming that gait speed cab be considered a measure of pre-frailty[25] and could be used as an early marker of health change among young older adults.

Heterogeneity in health increases with age. The number of different health states found in each age group increased from 27 among sexagenarians to 46 among nonagenarians. Greater heterogeneity in health status among older people, pointed out decades ago,[26] was confirmed recently by Lowsky^3^ in the US using survey data. We found that heterogeneity is particularly evident among nonagenarians, who have survived beyond the average life expectancy of their birth cohort, suggesting that multiple genetic and contextual factors are relevant to longevity, which can be achieved through a variety of pathways.[27]

Finally, our results confirm that several indicators of health are needed to characterize both the health status and the differences in need for medical, social, and hospital care among older people. Although age-related increases in all impairments were expected, we showed the differential capability of each indicator in capturing both intra- and inter-age health variations.

This study has both strengths and limitations. The SNAC-K participation rate was high, and we had the opportunity to estimate the effect of drop-outs. The study population covered a wide age range and included people with dementia and people living in institutions. Further, all participants were examined using standard procedures and criteria. However, the data are cross-sectional, so differences observed among age groups might be due to cohort effects and not only to changes associated with aging. We considered only five indicators of health. The indicators analyzed are objective reliable measures that are correlated with many other relevant health measures (e.g. polypharmacy). The population is also a selected group that has survived beyond baseline age requirements. Another limitation is selective participation in the younger age groups, as participants in these age groups were potentially healthier than those who declined to participate. The present study may thus underestimate the prevalence of poor health and overestimate homogeneity in people younger than 90 years. Finally, the educational level of the study population is higher than that in Stockholm or in Sweden. Although higher education level is associated with better functional status[28] it is also associated with longer survival,[29] which can results in higher occurrence of poor health.

## Conclusions

Our study provides a clear picture of heterogeneous health of older adults, which varies from good functioning, lack of disability, and no morbidity through morbidity and multimorbidity to severe disability. Most people younger than age 90 had functionally good health. We could identify two transitional periods: 1) 81–84, when prevalence of relatively good functional health decreased and prevalence of multimorbidity, lower cognitive functioning, and I-ADL disabilities increased and 2) 84–87, when higher prevalence of severe cognitive and physical impairment gradually led to disability in P-ADL. The first period seems to represent the passage from the third to the fourth age,[18] and the second, the beginning of the fourth age.[1] This means that the need for medical care increased from age 70 to 90, but the need for social assistance, including institutionalization, became prevalent only at very advanced ages.
