# Correction: Age-Related Variation in Health Status after Age 60

**DOI:** 10.1371/journal.pone.0130024

**Published:** 2015-06-03

**Authors:** Giola Santoni, Sara Angleman, Anna-Karin Welmer, Francesca Mangialasche, Alessandra Marengoni, Laura Fratiglioni

There are errors in the Author Contributions. The correct contributions are: Conceived and designed the experiments: GS SA AM LF. Analyzed the data: GS. Wrote the paper: GS. Revised paper: SA AKW FM AM LF
